# Eating fast is positively associated with general and abdominal obesity among Chinese children: A national survey

**DOI:** 10.1038/s41598-018-32498-9

**Published:** 2018-09-25

**Authors:** Xia Zeng, Li Cai, Jun Ma, Yinghua Ma, Jin Jing, Yajun Chen

**Affiliations:** 1 Department of Maternal and Child Health, School of Public Health, Sun Yat-Sen University, Guangdong, People’s Republic of China; 2 Institute of Child and Adolescent Health, School of Public Health, Peking University, Beijing, People’s Republic of China

## Abstract

Eating faster is related to more energy intake, but less is known about the relationships between children’s eating speed, food intake and adiposity, especially in high school children. This study aimed to investigate the associations of eating speed with general and abdominal obesity among Chinese children basing on a national survey. A total of 50,037 children aged 7–17 years were enrolled from 7 provinces in China in 2013. Anthropometric indices were objectively measured. Data on eating speed were collected by questionnaires. Increasing trends across the slow, medium, and fast eating speed group were observed in the prevalence of general obesity (7.2%, 10.0% and 15.9%), abdominal obesity (16.1%, 21.8%, and 29.4%) and waist-to-height ratio (WHtR) ≥ 0.5 (11.1%, 14.8%, and 22.0%). Compared with medium eating speed, fast eating speed was positively associated with obesity, abdominal obesity, and WHtR ≥ 0.5 (odds ratios [ORs]: 1.51~1.61), while slow eating speed was negatively associated with these outcomes (ORs: 0.65~0.75). Increased trends of consumption of fruits, meat/meat conducts, sugar-sweetened beverages, fried food, and fast food were observed in pace with increasing eating speed (*P* < 0.05). Our findings suggest that eating speed is positively associated with childhood general and abdominal obesity, which may be an important, modifiable factor to prevent childhood obesity.

## Introduction

Childhood obesity is increasingly common during the past several decades, leading to high disease burden^1^. In 2015, a total of 107.7 million children were obese worldwide^1^. China, a developing country with a population of over 1.3 billion, had experienced the rapid increase in prevalence of childhood overweight and obesity^2,3^, and had the highest number of obese children^1^. Therefore, addressing the epidemic of childhood obesity is of great significance, especially in China.

Behavioral and nutritional strategy plays a critical role in the prevention of obesity. Eating slowly, a crucial concept in behavioral nutrition, was recommended for weight management as it was confirmed to control satiety^4^. Such simple lifestyle change that may influence the individual risk for obesity is significant for cost-benefit prevention strategies. Recently, a systematic review included 23 studies and concluded that eating quickly was positively associated with excess body weight in adult populations^5^. The eating speed was evaluated using self-reporting in most of these studies (22/23) and using eating monitor in only one study; and the mean body mass index (BMI) or general obesity were used as outcome. Some other studies also suggested correlation between eating speed and childhood obesity^6–10^. Prospective study with a small sample also indicated that unhealthy eating behaviors (e.g. faster eating, overeating) will promote positive energy balance and then result in weight gain over a long time^11,12^.

However, gaps remain in the existing literature. First, previous studies mainly focused on a specific age group of children with small sample size, which precluded the opportunity to examine the age difference. Second, few studies with large sample sizes have been conducted to clarify the association between eating speed and abdominal obesity among children. BMI is perhaps the most commonly used measure for defining obesity in clinical practice, but it does not distinguish lean mass from fat mass^13^. Waist circumference (WC) and waist-height-ratio (WHtR), as proxy for abdominal fat mass, was also independent of BMI associated with the risk of mortality in adults, suggesting that abdominal obesity may be more strongly related to adverse metabolic outcomes^14–16^. Therefore, a practical and effective approach for the prevention of abdominal obesity is essential. Third, research has showed a positive relationship between eating speed and energy intake in pre-school children^10,17^, but whether energy or food intake vary by eating speed in older children has not been investigated.

Accordingly, using a population-based national study of children aged 7–17 years, the present study was to examine the relationship between eating speed and general and abdominal obesity. Moreover, we also examined the variation of food intake among different eating speed groups.

## Results

### Characteristics of participants

As presented in Table 1 of the 50,037 children, the mean (SD) age was 11.28 (3.07) years, and 51.1% were boys. The mean age and the proportion of boys increased significantly as the speed of eating increased. Regarding anthropometric characteristics, height, weight and WC increased significantly across the eating speed groups (slow, medium, and fast), and this trend was also observed in BMI and WHtR.

**Table 1:** Socio-demographic characteristics of participants by eating speed.

Variables	All	Eating speed			*P*
		Slow	Medium	Fast	
n	50037	11671	25613	12753	
Boys, %	51.1	46.0	48.5	60.9	**<0.001**
Age, (years)*	11.28 ± 3.07	10.85 ± 3.11	11.27 ± 2.98	11.68 ± 3.17	**<0.001**
Paternal educational level, %					**<0.001**
Primary school or below	7.5	7.2	7.2	8.2	
Junior high school	37.0	35.5	37.3	37.8	
High school	27.7	28.3	27.2	28.0	
Junior college or above	27.9	29.0	28.2	26.1	
Maternal educational level, %					**0.001**
Primary school or below	10.3	10.2	10.0	10.8	
Junior high school	38.2	36.8	38.5	38.8	
High school	26.1	26.7	25.8	26.2	
Junior college or above	25.4	26.3	25.6	24.2	
Family monthly income, %					**0.026**
≤2000, RMB	7.7	7.9	7.7	7.5	
2001~5000, RMB	24.9	25.5	24.5	25.2	
5001~7999, RMB	18.5	18.3	18.7	18.4	
≥8000, RMB	19.8	19.9	19.4	20.6	
No answer	29.0	28.3	29.7	28.2	
Family history of obesity, %	22.2	22.9	21.0	24.3	**<0.001**
Physical activity, (min, mean ± SE)					**<0.001**
Light	46.26 ± 0.31	44.32 ± 0.66	45.91 ± 0.42	48.71 ± 0.64	
Medium	28.31 ± 0.21	25.23 ± 0.41	28.65 ± 0.28	30.39 ± 0.45	
Vigorous	31.72 ± 0.22	27.85 ± 0.44	31.93 ± 0.31	34.66 ± 0.47	
Height, (cm)*	148.18 ± 15.89	144.69 ± 15.89	148.38 ± 15.43	150.99 ± 16.19	**<0.001**
Weight, (kg)*	42.68 ± 15.24	38.67 ± 13.83	42.46 ± 14.60	46.80 ± 16.64	**<0.001**
BMI, (kg/m^2^)*	18.83 ± 3.79	17.88 ± 3.44	18.73 ± 3.64	19.90 ± 4.11	**<0.001**
WC, (cm)*	65.86 ± 10.66	63.05 ± 9.84	65.70 ± 10.27	68.77 ± 11.40	**<0.001**
WHtR*	0.44 ± 0.05	0.436 ± 0.05	0.443 ± 0.05	0.460 ± 0.06	**<0.001**

*Values are shown as mean ± standard deviation.

Differences of age across eating speed was tested by using T-test, whereas differences of physical activity time, height, weight, body mass index (BMI), waist circumference (WC) and waist height ratio (WHtR) across different eating speed groups were tested by using analysis of covariance.

For categorical variables, differences of gender, parental and maternal educational, family history of obesity and family monthly income between eating speed groups were evaluated by using Pearson Chi-Square test.

### Associations of eating speed with general, abdominal obesity, and WHtR ≥ 0.5 by sex and age

As shown in Table 2, the prevalence of obesity, abdominal obesity and WHtR ≥ 0.5 among the participating children were 10.7%, 21.9% and 15.3%, respectively. The proportion of general obesity and WHtR ≥ 0.5 in boys (13.4%, 19.1%, respectively) was much bigger than in girls (7.7%, 11.3%, respectively), whereas prevalence of abdominal obesity in girls (22.5%) was bigger than in boys (21.3%). Increased trends were observed in the proportion of general and abdominal obesity, and WHtR ≥ 0.5 across different eating speed groups (slow, medium, and fast) in both boys and girls.

**Table 2:** The prevalence of obesity and abdominal obesity according to eating speed by gender.

Variables	All	Eating speed			*P for trend*
		Slow	Medium	Fast	
General obesity, %					
All	10.7	7.2	10.0	15.9	**<0.001**
Boys	13.4	9.5	12.4	18.7	**0.009**
Girls	7.7	5.4	7.8	11.5	**0.004**
Abdominal obesity, %					
All	21.9	16.1	21.8	29.4	**<0.001**
Boys	21.3	15.0	20.7	28.4	**<0.001**
Girls	22.5	17.1	22.8	31.1	**<0.001**
WHtR ≥ 0.5, %					
All	15.3	11.1	14.8	22.0	**<0.001**
Boys	19.1	14.0	18.6	25.3	**<0.001**
Girls	11.3	8.5	11.2	17.0	**<0.001**

All values are presented as percentage.

*Differences of obesity, abdominal obesity and WHtR ≥ 0.5 between eating speed groups stratified by gender were evaluated by using Chi-Square tests for trend.

Using multivariate logistic regression model, we analyzed the adjusted association between eating speed and obesity (Table 3). Eating fast (versus medium) showed positive association with obesity (adjusted odds ratio [OR]: 1.61, 95% confidential interval [CI]: 1.48–1.75), abdominal obesity (adjusted OR: 1.51, 95%CI: 1.41–1.61), and WHtR ≥ 0.5 (adjusted OR: 1.54, 95%CI: 1.43–1.65), whereas eating slow (versus medium) showed negative association with obesity (adjusted OR: 0.68, 95%CI: 0.61–0.75), abdominal obesity (adjusted OR: 0.70, 95%CI: 0.65–0.75) and WHtR ≥ 0.5 (adjusted OR: 0.71, 95%CI: 0.65–0.78). Similar associations of eating speed with general and abdominal obesity were observed in both sexes. The odds of eating fast for WHtR ≥ 0.5 was slightly higher in girls (OR: 1.62, 95%CI: 1.44–1.83) than that in boys (OR: 1.49, 95%CI: 1.39–1.63). In the age subgroup analyses, the significant associations of eating speed with obesity indices were also found in each age group, except that eating slow was not significantly associated with general obesity or WHtR ≥ 0.5 in the age group of 10–12 years. Similar results were found when we further adjustment for food intake (Supplementary Table S1).

**Table 3:** Associations of eating speed with obesity and abdominal obesity by gender and age.

Variables	Odd Ratio (95%CI)		
	Obesity	Abdominal obesity	WHtR ≥ 0.5
**Overall**			
slow	0.68 (0.61,0.75)**	0.70 (0.65,0.75)**	0.71 (0.65,0.78)**
medium	1.00	1.00	1.00
fast	1.61 (1.48,1.75)**	1.51 (1.41,1.61)**	1.54 (1.43,1.65)**
**Subgroups**			
**Slow**			
Boys	0.68 (0.59,0.78)**	0.69 (0.61,0.77)**	0.69 (0.61,0.77)**
Girls	0.65 (0.55,0.77)**	0.71 (0.64,0.78)**	0.75 (0.65,0.85)**
**Fast**			
Boys	1.64 (1.48,1.82)**	1.50 (1.37,1.64)**	1.49 (1.36,1.63)**
Girls	1.58 (1.37,1.81)**	1.52 (1.38,1.67)**	1.62 (1.44,1.83)**
**Slow**			
7~9 years	0.64 (0.55,0.74)**	0.66 (0.58,0.74)**	0.67 (0.59,0.77)**
10~12 years	0.84 (0.69,1.02)	0.83 (0.72,0.96)*	0.89 (0.76,1.05)
13~15 years	0.66 (0.50,0.86)*	0.65 (0.55,0.78)**	0.64 (0.51,0.79)**
16~17 years	0.33 (0.18,0.60)**	0.66 (0.51,0.85)**	0.57 (0.40,0.81)*
**Fast**			
7~9 years	1.70 (1.50,1.93)**	1.53 (1.37,1.70)**	1.57 (1.39,1.76)**
10~12 years	1.51 (1.29,1.77)**	1.42 (1.26,1.61)**	1.41 (1.23,1.61)**
13~15 years	1.61 (1.33,1.94)**	1.54 (1.35,1.76)**	1.61 (1.38,1.89)**
16~17 years	1.60 (1.19,2.14)*	1.62 (1.34,1.95)**	1.66 (1.32,2.08)**

Values are presented as OR and 95%CI. **P < 0.001, *P < 0.05.

Multivariate logistic regression model was applied with adjustment for gender, age, area, physical activity, family history of obesity, paternal and maternal educational level and family monthly income (all variables stratified by sex were not adjusted for gender).

### The dietary intake stratified by eating speed

Overall, children in the fast eating speed group tended to consume more fruit, meat/meat products and SSBs, as well as have a higher frequency of consuming fried food and fast food compared with those in the slow eating speed group (Table 4). In the contrary, the frequency of milk consumption was significantly less in the group of fast eating speed than that in the medium or slow eating speed group (P < 0.05). Similar findings were also observed in both sexes, especially in boys. In the total population, there was no significant difference of the vegetable or energy-dense snacks intake among different eating speed groups. However, results from subgroup analysis show that boys in the fast eating group were more likely to consume vegetable and fast food.

**Table 4:** The food frequency stratified by eating speed and gender.

Variables	Eating speed			*P*
	Slow	Medium	Fast	
**Overall**				
Fruit (servings/day)	1.23 ± 1.06	1.31 ± 1.07	1.34 ± 1.20	**0.015**
Vegetable (servings/day)	1.75 ± 1.42	1.85 ± 1.45	1.85 ± 1.46	0.925
Meat products (servings/day)	1.14 ± 1.17	1.14 ± 1.18	1.29 ± 1.33	**<0.001**
SSBs (cups/day)	0.39 ± 0.72	0.39 ± 0.73	0.50 ± 0.77	**<0.001**
Milk (days/week)	4.46 ± 2.61	4.54 ± 2.58	4.31 ± 2.70	**<0.001**
Energy-dense snacks (days/week)	2.10 ± 1.99	1.98 ± 1.92	2.00 ± 2.01	0.353
Fried food (days/week)	1.25 ± 1.59	1.20 ± 1.54	1.32 ± 1.70	**<0.001**
Fast food (days/month)	1.20 ± 2.22	1.18 ± 2.23	1.29 ± 2.50	**<0.001**
**Boys**				
Fruit (servings/day)	1.21 ± 1.10	1.29 ± 1.11	1.33 ± 1.23	**<0.001**
Vegetable (servings/day)	1.76 ± 1.47	1.86 ± 1.48	1.89 ± 1.50	**<0.001**
Meat products (servings/day)	1.27 ± 1.28	1.27 ± 1.29	1.43 ± 1.43	**<0.001**
SSBs (cups/day)	0.45 ± 0.82	0.48 ± 0.84	0.59 ± 0.89	**<0.001**
Milk (days/week)	4.49 ± 2.67	4.53 ± 2.63	4.37 ± 2.72	**<0.001**
Energy-dense snacks (days/week)	1.96 ± 1.95	1.91 ± 1.90	1.92 ± 2.00	**0.033**
Fried food (days/week)	1.23 ± 1.62	1.25 ± 1.59	1.34 ± 1.74	**0.005**
Fast food (days/month)	1.22 ± 2.36	1.20 ± 2.35	1.34 ± 2.66	**0.003**
**Girls**				
Fruit (servings/day)	1.25 ± 1.02	1.33 ± 1.04	1.34 ± 1.16	**<0.001**
Vegetable (servings/day)	1.75 ± 1.38	1.85 ± 1.41	1.80 ± 1.46	**<0.001**
Meat products (servings/day)	1.03 ± 1.06	1.02 ± 1.05	1.07 ± 1.12	**0.032**
SSBs (cups/day)	0.35 ± 0.61	0.31 ± 0.59	0.36 ± 0.68	**<0.001**
Milk (days/week)	4.44 ± 2.56	4.56 ± 2.54	4.21 ± 2.66	**<0.001**
Energy-dense snacks (days/week)	2.22 ± 2.03	2.05 ± 1.93	2.12 ± 2.02	**<0.001**
Fried food (days/week)	1.26 ± 1.56	1.16 ± 1.49	1.27 ± 1.63	**<0.001**
Fast food (days/month)	1.18 ± 2.08	1.16 ± 2.12	1.21 ± 2.56	0.393

*All *P* values were adjusted for age and area. Overall *P* values were also adjusted for gender.

## Discussion

Using a national representative sample, we clearly demonstrated positive associations of fast eating speed with general and abdominal obesity among children in both sexes and difference age groups. To the best of our knowledge, this is the first study to investigate these relationships in a child population including high school children. In addition, we found that fast eating speed was associated with a greater consumption of food except milk.

Previous studies have shown significant associations of fast eating speed with general obesity in children^10,18,19^, which was further supported by our study after adjustment for confounders. It was reported that WC and WHtR performed better than other anthropometric indices for the association with obesity-related health risk factors in children^20–22^. However, few studies described the relationships between eating speed and abdominal obesity, especially among children. In view of our results, fast eating speed was positively, and slow eating speed was negatively, associated with childhood abdominal obesity, even after further adjustment for food consumption and other variables. These findings are potentially of great public health significance. Randomized controlled trial has demonstrated modification of eating speed could be an efficient, cost effective adjunct for promoting weight management in obese^23^ and healthy adolescents^24^. A prospective study also confirmed that stopping eating quickly prevents excess weight gain in non-overweight/obese girls^25^ which could be helpful in fighting the global obesity epidemic. Nevertheless, there has been raised a problem that more than half of the target for slowing down of eating speed was unmet^26^. Therefore, instead of solely depending on individual willpower, strategies from different levels are needed to slow eating pace. For example, increasing the time available for school meals might be a practical and simple way to reduce excess food intake in school-aged children^27^.

Consistent with previous findings, we found that boys tend to have higher prevalence of general obesity and WHtR ≥ 0.5 in comparison with girls. This may be partly explained by the fact that boys were more likely to be eat faster than girls, as over 60% of faster eaters were boys in our study^9^. In addition, the association between eating speed and obesity indices in boys was similar in that in girls.

In the age subgroup analyses, we found that fast eating speed was positively linked with obesity indices, regardless of children’s age. One study reported that the strength of the above association was lower in children aged 12–15 years than in those aged 6–11 years^25^. However, these associations were relatively constant across the entire distribution of age in our study. Study, conducting among pre-school children aged from 3.1 to 6.7 years old, had also reported that eating speed was associated with overweight status^10^. These results have suggested that eating behaviors tend to be established early in life and effective strategies to slow down eating speed should be implemented from an early age.

The mechanisms underlying the relationships between fast eating speed and obesity are biological plausible. First, increasing amounts of food, prompted by fast eating speed, may increase total energy intake. Previous studies have showed that eating quickly led to higher energy intake but lower satiety^4,28^. The reason might be that a fast eating speed may interfere with the reflex signal that tells the body to stop eating when the stomach is full^29^. A very few studies have also demonstrated that children who ate fast had higher energy intake in a population level^6,10,19^. However, these studies only included pre-school children and have not assessed whether the association of eating speed and food intake is varied by food group. In the present study, we have shown that the consumption frequency of fruits, meat/meat conducts, SSBs, fried and fast food was significantly higher in children who ate faster, indicating more food and energy intake in this group. Interestingly, the frequency of milk consumption was the least in the group of fast eating speed, which may also increase the risk for obesity. Systematic reviews of clinical trials have summarized that increasing the intake of dairy foods under energy-restricted condition contribute to the reduction of individuals’ body weight and body fat^30,31^.

Second, eating slowly may benefit individuals by enhancing the thermic effect of food (TEF). It was concluded that eating slowly (eaten slowly >40 min V.S. eaten quickly >10 min) was associated with increased TEF, elevated serum adiponectin and suppressed endotoxin and nonesterified fatty acids in obese women^32^. A similar positive relationship between meal duration and post postprandial energy expenditure was also observed in healthy, normal weight individuals^33^. Third, eating speed may influence gastrointestinal satiety hormones. An intervention study undertaken in 27 obese adolescents have examined that eating more slowly has a significant impact on the gastrointestinal hormone (ghrelin, peptide tyrosine-tyrosine, pancreatic polypeptide, glucagon-like peptide-I, oxyntomodulin, and cholecystokinin), which control appetite and influence food consumption, suggesting that externally modifiable eating behaviors actually regulate the hormonal response to food^34^.

Several shortcomings should be acknowledged in our study. First, data on behavior factors, including eating speed, were collected using questionnaire, which may lead to report bias. The rate of eating is sometimes objectively evaluated by time spent eating a meal or using an eating monitor^35,36^, yet these methods are not necessarily representative of daily life, making subjective assessment by questionnaire much more widely used, especially in epidemiological settings^7,19,37^. In our study, the method of measuring eating speed, to some extent, might help avoid the impact of some confounding factors (e.g. age, gender, and geography). Furthermore, the cross-sectional nature of the study cannot determine the causality of the observed association. Moreover, the cluster sampling method applied in the recruitment of the participants might result in selection bias. Finally, we cannot deny the possibility that other potential confounding factors may mediate in the observed associations of eating speed with obesity.

## Conclusions

To summarize, we have shown that fast eating speed was positively associated with general and abdominal obesity among both girls and boys aged 7–17 years. Children who eat fast also had higher food intake except milk consumption. In the future, efforts are needed to better understand the influencing factors of eating fast and to develop approaches for slowing down the eating speed in populations.

## Methods

### Study design and participants

This was the baseline survey of a nationwide school-based intervention program targeted childhood obesity prevention in China (Registration number: NCT02343588)^38^. The survey was conducted between September and November 2013. This study complied with Declaration of Helsinki and was approved by the Ethical Committee of the Peking University. All participant students and their parents signed informed consents voluntarily.

Multistage random cluster sampling method was adopted in the recruitment of children and their parents. Firstly, all the seven geographic areas in China were chosen (i.e. central China, east China, south China, west China, northwest China, north China, and northeast China). One province/region was selected from each area as follows: Hunan, Shanghai, Guangdong, Chongqing, Ningxia, Tianjin, and Liaoning. Secondly, 12–16 primary and secondary schools (including junior high school and high school) were selected by probability proportional to size sampling method in each province/region (primary school: secondary school = 1:1). Thirdly, all students in grades 1 to 5, grade 7, grade 8, grade 10 and grade 11 were invited to participate. Other students were not selected because of their heavy study load. A total of 65,347 children and their parents were invited, 62,517 agreed to participate in the study. Children who had missing data on BMI (n = 5303), WC (n = 5632) or eating speed (n = 7177) were excluded, and the response rates of questionnaires were 100% (94/94) for schools and 87.4% (54,627/62,517) for students. Finally, 50,037 participants were included in the analyses.

### Questionnaire survey

The student and parent questionnaires were developed based on previously tested and validated questionnaires. To make sure that every question was explained similarly to all participants, the questionnaires were distributed to the whole class during school time by investigators who were trained postgraduates. The survey procedure in every class was supervised and assisted by head teachers. The questionnaires were completed by children together with their parents. School doctors uniformly collected all questionnaires from each class. Some investigators conducted quality check, and missing items were required to be filled out again and resubmitted.

### Student questionnaire

Children were asked about age, sex, eating speed, dietary behaviors and physical activities. Eating speed was assessed by asking children: Compared with your classmates with the same sex and age, how fast is your eating speed? The response options included slow, medium, or fast. Dietary intakes were obtained by using eight items. Children were asked to recall the frequency (days) and servings of fruits, vegetables, meat/meat products, and sugar-sweetened beverages (SSBs) during the past seven days. The average daily intakes of these food were calculated. Children also recalled the frequency of milk, energy-dense snacks, and fried food during the past seven days, as well as frequency of fast food intake during the last month. Vigorous-intensity physical activities, moderate-intensity physical activities, and walking were reported as days and duration in each of those days during the past week. The mean daily time spent in these activities were calculated.

### Parent questionnaire

A parent of each participant was asked about family history of obesity, paternal and maternal educational level (chosen from “primary school or below”, “junior high school”, “high school”, and “junior college or above”), and family monthly income (chosen from “<2000 RMB”, “2001~5000 RMB”, “5001~7999 RMB”, “≥8000 RMB”, and “refused to answer”).

### Anthropometric measurements

All anthropometric measurements were collected by the experienced technicians. Height (cm) was measured using Portable stadiometer to nearest 0.1 cm without shoes. Weight (kg) was measured using Lever type weight scale to nearest 0.1 kg with light cloths. WC was measured 1 cm above umbilicus to the nearest 0.1 cm in a standing position by a non-stretchable nylon tape. BMI was calculated by dividing body weight (kg) by the square of height (m^2^). Underweight, normal weight, overweight, and obesity (Obesity: BMI ≥ 95th percentile) were defined according to the age- and sex-specific BMI cut-offs for Chinese children^39^. The sex- and age-specific WC percentile was applied to determine abdominal obesity (≥90th)^40^. WHtR was calculated as WC(cm)/height(cm), and WHtR ≥ 0.5 was considered high WHtR^22^.

### Statistical analysis

Continuous and categorical variables were presented as mean and standard deviation (SD), and percentage, respectively. For continuous variables, difference of age, physical activities time, height, weight, BMI, WC and WHtR across eating speed categories were tested by using analysis of covariance. For categorical variables, differences of sex, parental and maternal educational level, family history of obesity, and family monthly income across eating speed groups were evaluated by using Pearson Chi-Square test. Differences of the prevalence of obesity, abdominal obesity and WHtR ≥ 0.5 among eating speed groups stratified by sex were evaluated by using Chi-Square tests for trend. Multivariate logistic regression models were used to examine the associations of eating speed with general obesity, abdominal obesity, and WHtR ≥ 0.5, with the medium eating speed group as reference. Covariates selected a priori included sex, age, area, physical activity, family history of obesity, paternal and maternal educational level, and family monthly income. The dietary intake was calculated by eating speed, and age-, sex- and area-adjusted difference across different eating speed group was tested by using analysis of covariance. All statistical analysis was performed using IBM SPSS software version 21.0. A two-tailed P < 0.05 was considered statistically significant in all analysis.

### Ethical approval and informed consent

1. The project was approved by the Ethical Committee of Peking University.
2. Informed consent forms were obtained from all participating students and their parents.

## Electronic supplementary material

Table S1
