# Supplementary material for: Eating fast is positively associated with general and abdominal obesity among Chinese children: A national survey
Source: Sci Rep. 2018 Sep 25;8:14362. doi: 10.1038/s41598-018-32498-9 (PMC6156407; doi:10.1038/s41598-018-32498-9)
Supplement: Supplementary file 1 — Table S1 [file 41598_2018_32498_MOESM1_ESM.docx]

**Eating fast is positively associated with general and abdominal obesity among Chinese children: A national survey.**

**Xia Zeng ^1, +^, Li Cai ^1, +^, PhD, Jun Ma ^2^, PhD, Yinghua Ma ^2^, PhD, Jin Jing ^1, *^, MD, PhD, and**

**Yajun Chen ^1, *^, PhD**

1: Department of Maternal and Child Health, School of Public Health, Sun Yat-Sen University, Guangdong, People’s Republic of China.

2: Institute of Child and Adolescent Health, School of Public Health, Peking University, Beijing, People’s Republic of China.

***Corresponding author**

chenyj68@mail.sysu.edu.cn (YJ Chen); jingjin@mail.sysu.edu.cn (J Jing);

+ these authors contributed equally to this work

**Supplementary Table S1 Associations of eating speed with obesity and abdominal obesity by gender and age**

| Variables | Odd Ratio (95% CI) | | |
| --- | --- | --- | --- |
|  | Obesity | Abdominal obesity | WHtR≥0.5 |
| **Overall** |  |  |  |
| slow | **0.67 (0.60,0.75) **** | **0.71 (0.65,0.77) **** | **0.72 (0.66,0.79) **** |
| medium | 1.000 | 1.000 | 1.000 |
| fast | **1.63 (1.49,1.78) **** | **1.50 (1.40,1.61) **** | **1.53 (1.42,1.65) **** |
|  |  |  |  |
| **Subgroups** |  |  |  |
| **Slow** |  |  |  |
| Boys | **0.68 (0.59,0.79) **** | **0.69 (0.61,0.78) **** | **0.69 (0.61,0.78) **** |
| Girls | **0.65 (0.54,0.77) **** | **0.73 (0.66,0.81) **** | **0.76 (0.66,0.88) **** |
| **Fast** |  |  |  |
| Boys | **1.65 (1.48,1.84) **** | **1.48 (1.35,1.63) **** | **1.46 (1.33,1.61) **** |
| Girls | **1.57 (1.35,1.82) **** | **1.54 (1.40,1.70) **** | **1.64 (1.45,1.85) **** |
|  |  |  |  |
| **Slow** |  |  |  |
| 7~9 years | **0.67 (0.570,0.78) **** | **0.60 (0.61,0.78) **** | **0.69 (0.60,0.79) **** |
| 10~12 years | 0.85 (0.69,1.05) | **0.82 (0.71,0.96) **** | 0.90 (0.76,1.067) |
| 13~15 years | **0.60 (0.44,0.81) **** | **0.65 (0.54,0.78) **** | **0.65 (0.52,0.82) **** |
| 16~17 years | **0.30 (0.16,0.54) **** | **0.66 (0.51,0.86) **** | **0.51 (0.36,0.74) **** |
| **Fast** |  |  |  |
| 7~9 years | **1.76 (1.55,2.01) **** | **1.54 (1.37,1.72) **** | **1.55 (1.37,1.756) **** |
| 10~12 years | **1.46 (1.23,1.73) **** | **1.45 (1.27,1.66) **** | **1.43 (1.22,1.64) **** |
| 13~15 years | **1.54 (1.26,1.87) **** | **1.49 (1.30,1.71) **** | **1.57 (1.33,1.84) **** |
| 16~17 years | **1.64 (1.22,2.21) **** | **1.56 (1.28,1.90) **** | **1.61 (1.28,2.03) **** |

Values are presented as OR and 95% CI. ***P*<0.001, **P*<0.05.

Multivariate logistic regression model was applied with adjustment for food consumption, gender, age, area, physical activity, family history of obesity, paternal and maternal educational level and family monthly income (all variables stratified by sex were not adjusted for gender and stratified by age were not adjusted for age.)
